# Refocusing on sexually transmitted infections (STIs) to improve reproductive health: a call to further action

**DOI:** 10.1186/s12978-021-01296-4

**Published:** 2021-12-01

**Authors:** Akaninyene Otu, Georges Danhoundo, Igor Toskin, Veloshnee Govender, Sanni Yaya

**Affiliations:** 1 Department of Infection and Travel Medicine, Leeds Teaching Hospitals NHS Trust, Leeds, UK; 2 Department of Internal Medicine, College of Medical Sciences, University of Calabar, Calabar, Cross River State Nigeria; 3 Department of Sexual and Reproductive Health and Research, World Health Organization, Geneva, Switzerland; 4 School of International Development and Global Studies, University of Ottawa, 120 University Private, Ottawa, ON K1N 6N5 Canada; 5 The George Institute for Global Health, Imperial College London, London, UK

## The current state of sexually transmitted infections (STIs)

STIs are infections that result from the transmission of a pathogenic organism by sexual contact [1]. Globally, STIs constitute a major burden and a significant public health challenge, driven by health systems challenges related to inadequately resourced, poor quality services on the one hand and social stigma and discrimination surrounding STIs on the other, which together discourage many people from seeking and receiving services (i.e. counselling, testing, and treatment) [2]. The World Health Organization (WHO) estimated that in 2016 the global annual incidence of four of the eight most prevalent STIs (i.e. chlamydia, gonorrhoea, trichomoniasis and syphilis) among people 15–49 years of age was 376.4 million infections [3]. These four infections are curable, unlike viral infections such as HIV, hepatitis B, herpes simplex virus (HSV) and human papillomavirus (HPV), requiring long term management and treatment.

## Intersection between sexual health, reproductive health and STIs

Sexual health, which encompasses STIs, is understood as a state of physical, emotional, mental and social wellbeing in relation to sexuality. It encompasses not only certain aspects of reproductive health—such as being able to control one’s fertility and being free from sexual dysfunction and sequelae related to sexual violence or female genital mutilation—but also, the possibility of having pleasurable and safe sexual experiences, free of coercion, discrimination and violence [4].

Sexual health and reproductive health are inextricably interlinked. For example, efforts to prevent and control chlamydia have important implications for future fertility, as chlamydia is a major cause of infertility. Likewise, access to and use of contraception can impact sexual pleasure and enjoyment [5].

Despite significant strides made globally in improving reproductive health, 180 women are estimated to die daily from complications of pregnancy and childbirth arising mostly from preventable or treatable causes, such as infections [6]. STIs account for a significant proportion of infections that impact negatively on reproductive health. Major disruptions to health services arising from the COVID-19 pandemic has further aggravated the tenuous situation, particularly for vulnerable groups globally and deepening inequities in access and health outcomes [7]. These vulnerable groups include the youth, young women and migrants. Given the unequivocal recognition that reproductive health (and sexual heath) are a cornerstone of sustainable development, global agencies including the World Health Organization (WHO) and the United Nations Population Fund (UNFPA) have remained resolute in their call for the reprioritisation of reproductive health [7, 8]. This has culminated in the launch of the Ending Preventable Maternal Mortality (EPMM) initiative by the WHO and UNFPA with established targets to be achieved by 2025 [6].

The reproductive health consequences of STIs include pelvic inflammatory disease with permanent damage to the fallopian tubes, uterus, and surrounding tissues, which can lead to infertility, mother-to-child transmission, adverse pregnancy outcomes and chronic pelvic pain. For example, STIs are estimated to cause infertility in at least 24,000 women each year in the US [9]; over 900,000 pregnant women were confirmed to have syphilis in 2012 and this resulted in approximately 350,000 adverse birth outcomes including stillbirth. In 2016, there were an estimated 473 congenital syphilis cases per 100,000 live births [10].

STIs can reduce fertility of the infected individuals via several mechanisms: by directly damaging organs and reproductive cells, by indirectly inducing tissue damage, scarring and obstruction [11, 12]. In 2020, the estimated incident cases of STIs globally were as follows: 128 million chlamydia cases; 82 million gonorrhoea cases; 7.1 million trichomoniasis cases and 7.1 million syphilis cases [13]. Alarming increases both in gonorrhoea and syphilis is being recorded among men who have sex with men (MSM) and bisexual men [14] coupled with the increasing global incidence of drug-resistant gonorrhoea even in affluent countries such as the US [15].

HPV remains the most common viral infection of the reproductive tract with two HPV types (16 and 18) giving rise to 70% of cervical cancers and pre-cancerous cervical lesions [16]. In 2020 alone, there were a total of 604,000 new cases of cervical cancer with 341,000 deaths from cervical cancer and 1.5 million people were newly infected with hepatitis B virus [17]. HPV and hepatitis B are STIs which are preventable by vaccination. The presence of an STI magnifies the risk of acquiring or transmitting HIV by as much as two to three times [18]. HIV continues to have devastating health effects globally, having killed 36.3 million people so far with an estimated 37.7 people living with HIV at the end of 2020, over two thirds (25.4 million) of whom are in Africa [19].

## Giant strides made

In 2016, the WHO proposed five strategic directions for 2016–2021 with priority actions for countries to prevent and control STIs [18]. The strategic directions were in line with the 2030 Agenda for Sustainable Development for ending STIs as a major public health issue and included: information for focused action (understanding the STI epidemic and response); interventions for impact (addressing the first dimension of universal health coverage); delivering for equity (addressing the second dimension of universal health coverage); financing for sustainability and innovation for acceleration. The focus of these strategies was primarily on three key STIs namely gonorrhoea (in view of the risk of coinfection with other STI’s and rising risk of drug-resistant gonorrhoea), syphilis (with the aim of eliminating congenital syphilis), and HPV (with a focus on vaccination to eliminate cervical cancer) [18].

Significant progress has been made with the establishment of screening and monitoring mechanisms for STIs in many countries. By 2019–2020, 97 of 110 countries (87%) surveyed by the WHO had STI surveillance in place with 64% of countries having conducted surveillance of gonococcal antimicrobial susceptibility in 2019–2020 [18]. In the same time period, 19 (25%) of 75 countries surveyed reported > 80% HPV vaccine coverage [13]. With respect to HIV and hepatitis B diagnosis, an estimated 84% of people living with HIV were aware of their HIV status in 2020 [20] while 30.4 million people living with hepatitis B knew their hepatitis B status at the end of 2019.

Advances have also been recorded with improved access to treatment of STIs. The majority of countries (103 of 111; 93%) surveyed had policies for antenatal screening and treatment of syphilis in place in 2019–2020. Moreover, 59% of countries included the HPV vaccine in the national immunization schedule [18]. In 2020, 73% of people living with HIV accessed treatment and 6.6 million people with hepatitis B received treatment in 2019 [18].

## Yet many gaps remain

Despite many countries having established screening and treatment programmes for STIs, the incidence of most STIs such as chlamydia, gonorrhoea and trichomoniasis is plateauing [21]. The underlying reasons, while context-specific, are largely combination of both health systems and service delivery challenges as well as wider social and gender norms that contribute to stigma and discrimination in both communities and healthcare settings.

On the health systems side, across many settings, insufficient integration of STI services with existing and routine services (e.g. family planning, maternal health, HIV) at primary care challenges both effective service and continued reliance on vertical programmes contributes to duplication of services and challenges access and overall sustainability [21]. In addition, lack of trained health workers, laboratory capacity, adequate supplies of appropriate medicines and capacity to screen for asymptomatic infections contribute to poor quality services.

For STIs such as hepatitis B, treatment is lifelong and requires regular laboratory monitoring of hepatitis B viral load, which adds to the overall cost of controlling such an STI. However, there is evidence that testing and treatment for STIs remain cost-effective interventions [22]. Despite this, there is a seeming reluctance on the part of many governments to fund and execute STI control strategies. These issues have been given lower priority in the face of competing demands for scarce resources [23].

On the demand side, social and gender inequalities, stigma and discrimination disproportionately affect vulnerable and key populations, both in terms of risk of infection and access to STI services. These populations include adolescent girls and young women, sex workers, MSM, people who inject drugs, prisoners and transgender people [21]. In low- and middle-income countries, which account for 75–80% of STIs, poor access to effective and affordable STI services and high prevalence of drug resistant pathogens is disproportionately experienced by young people who are sexually active; migrant and displaced due to wars and natural catastrophes; people involved in transactional sex [22].

## What is left to be done?

Globally, reproductive health cannot be improved without urgently accelerating progress and delivering on commitments to existing STI control strategies. Primary prevention of STIs should be upscaled to modify sexual behaviour by encouraging practices such as delayed onset of sexual debut, sexual abstinence and mutually faithful sexual relationships among communities as well as promotion of partners reduction and condom use. Long-term outcomes of primary prevention strategy should include a paradigm shift from a disease-centered approach to sexual health and reproductive health promotion with a positive attitude towards sexuality, based on the core concept of well-being, and tailored behavioural interventions linked to the evidence-based biomedical approaches in one package to prevent new cases of STIs [24].

Promotion of appropriate treatment seeking behaviour for STIs should be promoted at grassroots level. Provision of treatment of people with STIs in order to prevent further transmission among communities remains a key strategy to eliminate STIs as a public health threat. To achieve this, STI prevention and care services will need to be cascaded to the primary health care level. Intensified screening and case finding of STI should be widely adopted along with linkage to treatment and engagement in care. Vaccination activities for hepatitis B and HPV will need to be expanded by carefully addressing policy bottlenecks, cost, vaccine supply and rollout issues. Capacity building (improvement of health provider competencies) should be implemented for a wide range of actors in healthcare to support this process.

To facilitate the provision of quality and equitable STI care services at grassroots level, the WHO launched updated guidelines for the management of symptomatic STIs in June 2021 [21]. These guidelines which provide updated, evidence-informed clinical and practical recommendations for managing persons with STIs should be adopted and implemented at scale.

Targeted STI control interventions for vulnerable and key populations such as adolescent girls, MSM and people who inject drugs should be prioritised. With the current push towards elimination of some of these STIs, price reduction strategies for STI testing, vaccines and drugs are critical. More research is essential to discover safe, effective and cheaper vaccines, drugs and point of care tests for STIs and address the emerging antibiotic resistance. Greater allocation of funds for STI control activities is required from government and relevant stakeholders. Private–public partnership in the provision of STI testing and treatment should be facilitated to promote resource sharing, collaboration and economies of scale.

STIs pose behavioural, social, economic, structural, and political challenges—these multifaceted issues should be addressed by interventions at multiple points along the STI-care continuum via a combination of prevention strategies, backed by strong political and financial commitment. Only by priority setting and multisectoral public health approaches will the STI epidemic be addressed and reproductive health and rights guaranteed.
